# Bisphenol A environmental exposure and the detrimental effects on human metabolic health: is it necessary to revise the risk assessment in vulnerable population?

**DOI:** 10.1007/s40618-015-0336-1

**Published:** 2015-06-24

**Authors:** R. Valentino, V. D’Esposito, F. Ariemma, I. Cimmino, F. Beguinot, P. Formisano

**Affiliations:** Institute of Experimental Endocrinology and Oncology (IEOS), National Council of Research (CNR) IEOS-CNR, Federico II University of Naples, via Pansini, 5, 80131 Naples, Italy; Department of Translational Medical Sciences, Federico II University of Naples, via Pansini, 5, 80131 Naples, Italy

**Keywords:** Endocrine disruptors, Bisphenol A, Prenatal/neonatal exposure, Low-grade-inflammation, Obesity, Insulin resistance

## Abstract

In the last decades, many reports have focused the attention on deleterious effects of novel environmental chemical compounds, including bisphenol A (BPA), on human health. BPA, a common and widely chemical contaminant acting as endocrine disruptor, accumulates in adipose tissue and may affect adipocyte metabolic and inflammatory functions. BPA, at low chronic doses, is now considered as an obesogen compound, and might contribute to the rise of metabolic syndrome, visceral adiposity and diabetes epidemics. The BPA worldwide presence in the environment is responsible for chronic exposure during vulnerable periods, such as foetal and neonatal life. The BPA source of contamination can occur via food, beverage, wastewater, air, dust and soil. BPA, as lipophilic compound, may accumulate into the adipose tissue already during foetal life and may affect adulthood health, through adverse effects on the growth and development of organs and tissues. Thus, based on several studies, it would be crucial to consider further actions aimed to refine risk assessment at least in vulnerable population, such as foetuses, infants and young children, to prevent metabolic diseases and obesity.

## Introduction

Recent advances have confirmed the role of chemical environmental pollutants, together with excessive nutrients, in the global obesity epidemic, mainly via adipose tissue dysfunction and inflammation. In particular, these pollutants interfere with several function of endocrine system and so they are called endocrine disrupting chemicals (EDC). Among these compounds, bisphenol A (BPA), primarily used to make polycarbonate plastic and epoxy resins, could represent a causative link between chemical exposure and the “obesogen hypothesis” [1, 2, 4]. BPA is an ubiquitous lipophilic compound with oestrogenic activity and it is able, for example, to migrate from can coatings into foods and beverages during storage, depending on heating process, contact with oil or acetic acid [1, 3].

Human BPA exposure is controversially discussed in non-occupational subjects. For its worldwide chronic presence, BPA is detectable in human serum, urine, amniotic fluid, placental tissue and umbilical cord blood [4, 5]. The total number of epidemiological and toxicological studies in humans are still very small. Several researches, involving in vitro and in vivo models, including humans, wildlife species and laboratory animals, were focused on the mechanistic basis of BPA action in different experimental conditions [1, 3–5]. Current limitations in understanding the global consequences of BPA exposure include incomplete understanding of the cell/tissue specific actions and effects, particularly at different critical windows of susceptibility during the entire life-span [1, 3–6].

### BPA environmental chronic exposure and biological effects

A modified synthesis of the most relevant sources of BPA reported in literature has been shown in Table 1, with the range of concentrations in general environment, including water (river and sea), air and dust [5]. Of interest, BPA human daily intake occurs mostly via foods and beverages, but also via skin exposure after continuously handling thermal paper receipts in occupationally exposed populations such as cashiers, who handle receipts 40 or more hours per week [7].

**Table 1:** Illustrating the most relevant sources of BPA and its levels in environment, food, beverage, containers and other origins (modified by Kang et al. [5])

BPA contamination sources	BPA concentration (range)
Aquatic environment	8–21 ng/ml
Air	2–208 ng/m^3^
Dust	0.8–10 μg/g
Thermal paper	54–79 μg/cm^2^
Meats	17–602 ng/g
Fish	5–109 ng/g
Vegetables and fruits	9–76 ng/g
Beverages	1–18 ng/g
Dairy products	21–43 ng/g
Infant formula	0.1–13 ng/g
Cans	2–82 ppb
Plastics	0.2–26 ppb
Dental materials	0.013–30 mg

*ppb* parts per billion

**Fig. 1:**
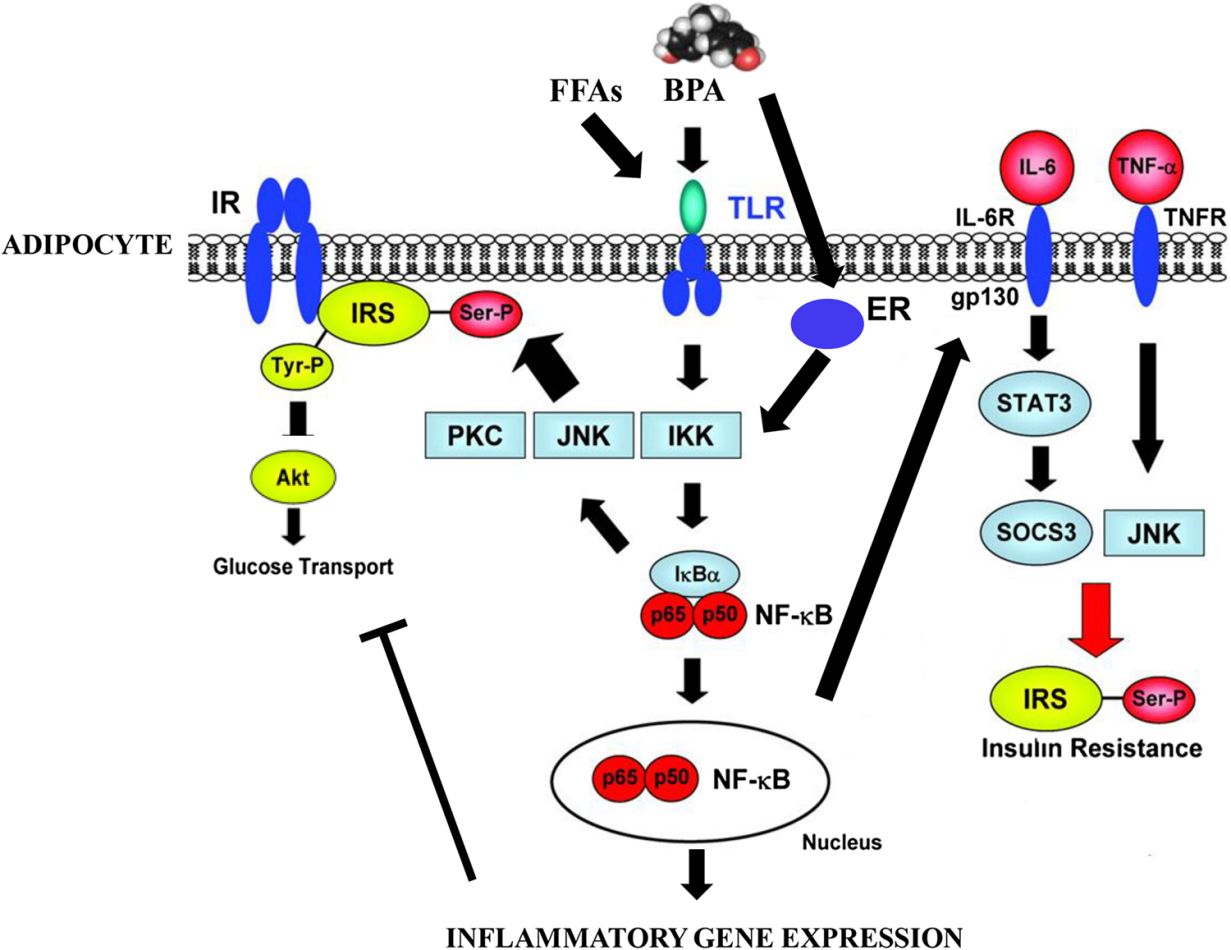
Putative scheme on bisphenol A (BPA) action in adipocyte. BPA can bind oestrogen receptors and/or TLR/cytokine receptors. BPA may modulate cytokine synthesis by activation of JNK/STAT3/NFkB inflammatory pathway, and by influencing insulin network via IR/AKT down-regulation

In humans, BPA chronic exposure, even at nanomolar concentrations, has been reported to be deleterious, mainly involving human reproduction, development, metabolic and inflammatory pathways [1, 2, 4–6]. The number of papers published on PubMed between 1975 and 2015 has been shown in Tables 2 and 3, reporting the main negative effects of BPA, that involve human and animal in vitro and in vivo studies, respectively. Several analyses, including human epidemiological studies, have supported the BPA “obesogen” effects [1, 3–6, 8]. For instance, we have recently reported a significant activation of inflammatory pathways (JNK, STAT3 and NF-kB), with an increased secretion of pro-inflammatory cytokines (IL-6 and INF-γ) in human adipocytes, incubated in vitro with 1 nM BPA for 24 h. This pro-inflammatory effect is associated with down-regulation of the insulin signalling network and inhibition of insulin-stimulated glucose utilization [9]. Moreover, these results were confirmed in a cross-sectional study on a male population, where a significant correlation was found between BPA plasma levels, inflammation and visceral obesity [8]. In this context, it has been also reported a link between BPA plasma levels, markers of low-grade inflammation and insulin resistance in polycistic ovary syndrome [10], an enigmatic syndrome in which experimental exposure to industrial EDC further deteriorate the hormonal and fertility imbalances [11]. Thus, a putative mechanism by which BPA affects adipocyte metabolic functions and leads to insulin resistance, probably via inflammatory pathways has been hypothesized (Fig. 1).

**Table 2:** Illustrating in vitro studies showing the main negative effects of BPA, involving different tissue and cell models

In vitro studies	Cell models	Negative effects	Number of published papers (PubMed 1975–2015)
Adipose tissue	3T3L1 preadipocytes and mature adipocytes SVF and human adipocytes	Adipocyte differentiation Insulin signaling Inflammatory effects	6
Bone tissue	MC3T3-E1 osteoblastic-like Reticulocytes	Cell proliferation Cell differentiation Ca^2+^ content	8
Breast cancer	MCF7 T47D BT20 MDA-MB231	Cell proliferation Cell growth ERE-promoter activation DNA damage	27
Immune system	Primary splenocytes (mice) Primary neutrophil granulocytes	Cytokines production Reactive species Intracellular Ca^2+^ Th1 and Th2 responses	91
Liver	HEP3B	Hypoxic response Lipid accumulation	50
Reproductive system	TTE3 sertoli cells Spermatogenic cells LNCaP (prostate) Granulosa and theca cells	mRNA expression AR-ER transactivation AR-ER expression	31
Nervous system	Primary culture midbrain astrocytes Neuron/glia	Cell differentiation GFAP/NeuN immunoreactivity	47
Pancreas	Primary pancreatic islets Beta-cells	Insulin release and content Intracellular Ca^2+^ flux Apoptosis	3

All data were extrapolated from “draft scientific opinion (EFSA panel 2015)” and from PubMed revision from years 1975 to 2015, reported as number of published papers

*ERE* oestrogen response element, *AR-ER* androgen receptor-oestrogen receptor, *GFAP/NeuN* glial fibrillary acidic protein/neuron-specific nuclear protein

**Table 3:** Illustrating in vivo studies showing the main negative effects of BPA, involving different models

	Methods	Negative effects	Number of published papers (PubMed 1975–2015)
In vivo human studies	Biomonitoring methods Correlation studies Cross-sectional studies Plasma/urine determinations (ELISA, GC/MS,HPLC, LC/MS)	General toxicity Reproduction and development Neurodevelopment and neuroendocrine system Immune system Cardiovascular system Metabolism (diabetes, weight gain, obesity) Genotoxicity Carcinogenicity	442
In vivo animal studies (mice, rats, monkeys)	Prenatal exposure Neonatal exposure Adult animals	Immune system Metabolism (glucose intolerance, hyper- insulinaemia, diabetes, weight gain, hyper- tryglyceridemia) Cancer Reproduction and development	565

All data were extrapolated from “draft scientific opinion (EFSA panel 2015)” and from PubMed revision from years 1975 to 2015, reported as number of published papers

Besides these well-documented molecular mechanisms on adipose tissue, inflammation and glucose metabolisms, several biological effects have been attributed to low-dose environmental BPA exposure [1, 3–6] (Tables 2, 3). Due to its ability to mimic oestrogen activity, BPA can enhance or inhibit the activity of endogenous oestrogens, mainly acting via nuclear oestrogen receptors ERα and ERβ, but also binding G-protein coupled oestrogen receptor (GPR30). Moreover, BPA could compete with 5α-dihydrotestosterone (DHT) for binding at the androgen receptor (AR) causing an anti-androgen effects, leading to male infertility, but also at thyroid hormone receptor (TR), with an interference on TR, affecting the brain morphology and expression of genes related to brain development. Another interesting BPA effect is on pancreatic beta-cells, where BPA can activate receptor-mediated pathways, influencing the rapid influx of calcium and increasing insulin release and glucose metabolism. On the immune system, BPA can affect the immune system functions in humans, involving autoimmunity, adaptative immune response, such as Th1 and Th2 responses, but also elicits differential responses on cytokine production, cell-type and stimulus specific [1, 3–6].

### BPA and foetal exposure

The detrimental effects of BPA on tissue and organ development during foetal life are now well documented in literature, mainly in animal models (Table 2). In humans, detectable BPA plasma and urine levels have been reported in several epidemiological studies, with higher values in children and consistent associations with metabolic alterations [1, 3–6]. As consequence of the BPA worldwide presence and to its ability to cross placenta, humans, during the whole life, including foetal and neonatal life, are chronically under BPA low doses exposure. Interestingly, when ingested, BPA is rapidly absorbed mainly at gastrointestinal level and partially inactivated in the liver and intestine via conjugation by uridine 5′-diphospho-glucuronosyl-transferase (UDP-UGT) activity [1, 3–6]. Foetal liver has an immature system of detoxifying chemicals, in particular UDP-UGT activity is reduced, and the embryonic/foetal BPA exposure, with subsequently accumulation, may impair the tissue embryonic development and function, particularly affecting adipose tissue functions, insulin sensitivity and fat increase [6, 12]. Moreover, these findings raise the possibility that BPA may alter regulatory genes of the adipogenesis process, at least in part by epigenetic mechanisms.

It should be noticed that BPA doses, considered “safe” by the European Food Safety Authority (EFSA) [13], may reach “internal” exposure levels higher than those usually required to stimulate molecular endpoints in cultured cells [1, 2, 4–7]. Indeed, BPA at environmental doses may be responsible for reprogramming of the endocrine-metabolic status, with development of systemic low-grade inflammation, insulin resistance and metabolic syndrome during the whole lifespan, particularly when other environmental challenges occur, such as high-fat diet [1–6, 8, 9, 12].

Considering the significant difference in BPA pharmacokinetic between animals and humans and in low-dose vs high-dose effects, additional in vivo and in vitro studies involving humans are needed to better define BPA metabolic effects. In particular, BPA deleterious effects should be better defined in vulnerable population, such as foetuses, infants and young children, where also cumulative with mixture of different environmental chemical pollutants may affect the correct tissue development and organogenesis. The embryonic critical window of exposure, can involve systemic metabolic homeostasis and adulthood health programming, in agreement with the new concept of “developmental origins of health and disease”, which underlies foetal basis for adult diseases [12].

In other words, BPA foetal/neonatal exposure levels, which may be responsible for increased human health risk, should not be underestimated. Current researches are in progress to sort the effect of foetal programming as a contributing factor in adult metabolic diseases, particularly in obesity, insulin resistance, metabolic syndrome and diabetic epidemics [6, 12].

Thus, although all of these hypotheses need thorough examination, caution should be given by National and International Regulatory Agencies, on the potential deleterious role of BPA [14] but also of BPA-like compounds (such as bisphenol F and bisphenol S), which are gradually replacing BPA in industrial applications [15].

## Conclusions

In humans, the continuous BPA chronic exposure from canned goods, drinking plastic packaging and thermal paper must be reduced. To avoid the contact between plastic containers and food and beverage, particularly during foetal/neonatal life, must be the future goal, for example by privileging fresh foods. Interestingly, France has recently abolished BPA from all packaging food, to reduce drastically the BPA intake. Safety guidelines should give more attention to the potential consequence in vulnerable people worldwide, also considering that the BPA environmental exposure is modifiable through behavioural changes. A reduction of BPA exposure can represent an attractive target for prevention of diseases which represent a serious problem of global proportion and economic and social emergencies, such as obesity, metabolic syndrome and diabetes.

## References

[1] Pereira-Fernandes A, Demaegdt H, Vandermeiren K, Hectors TL, Jorens PG, Blust R, Vanparys C (2013). Evaluation of a screening system for obesogenic compounds: screening of endocrine disrupting compounds and evaluation of the PPAR dependency of the effect. PLoS One.

[2] Vandenberg LN, Hauser R, Marcus M, Olea N, Welshons WV (2007). Human exposure to bisphenol A (BPA). Reprod Toxicol.

[3] Wetherill YB, Akingbemi BT, Kanno J, McLachlan JA, Nadal A, Sonnenschein C, Watson CS, Zoeller RT, Belcher SM (2007). In vitro molecular mechanisms of bisphenol A action. Reprod Toxicol.

[4] Rochester JR (2013). Bisphenol A and human health: a review of the literature. Reprod Toxicol.

[5] Kang JH, Kondo F, Katayama Y (2006). Human exposure to bisphenol A. Toxicology.

[6] Liu J, Yu P, Qian W, Li Y, Zhao J, Huan F, Wang J, Xiao H (2013). Perinatal bisphenol A exposure and adult glucose homeostasis: identifying critical windows of exposure. PLoS One.

[7] Ehrlich S, Calafat AM, Humblet O, Smith T, Hauser R (2014). Handling of thermal receipts as a source of exposure to bisphenol A. JAMA.

[8] Savastano S, Tarantino G, D’Esposito V, Passaretti F, Cabaro S, Liotti A, Liguoro D, Perruolo G, Ariemma F, Finelli C, Beguinot F, Formisano P, Valentino R (2015). Bisphenol-A plasma levels are related to inflammatory markers, visceral obesity and insulin-resistance: a cross-sectional study on adult male population. J Transl Med.

[9] Valentino R, D’Esposito V, Passaretti F, Liotti A, Cabaro S, Longo M, Perruolo G, Oriente F, Beguinot F, Formisano P (2013). Bisphenol-A impairs insulin action and up-regulates inflammatory pathways in human subcutaneous adipocytes and 3T3-L1 cells. PLoS One.

[10] Tarantino G, Valentino R, Di Somma C, D’Esposito V, Passaretti F, Pizza G, Brancato G, Orio V, Formisano P, Colao A, Savastano S (2013). Bisphenol A in polycystic ovary syndrome and its association with liver-spleen axis. Clin Endocrinol.

[11] Palioura E, Diamanti-Kandarakis E (2013). Industrial endocrine disruptors and polycystic ovary syndrome. J Endocrinol Invest.

[12] Alonso-Magdalena P, García-Arévalo M, Quesada I, Nadal Á (2015). Bisphenol-a treatment during pregnancy in mice: a new window of susceptibility for the development of diabetes in mothers later in life. Endocrinology.

[13] European Food Safety Authority (EFSA) (2015) Report on the two-phase public consultation on the draft EFSA scientific opinion on bisphenol A (BPA). EFSA supporting publication EN-740

[14] Myers JP, Vom Saal FS, Akingbemi BT, Arizono K, Belcher S, Colborn T, Chahoud I, Crain DA, Farabollini F, Guillette LJ, Hassold T, Ho SM, Hunt PA, Iguchi T, Jobling S, Kanno J, Laufer H, Marcus M, McLachlan JA, Nadal A, Oehlmann J, Olea N, Palanza P, Parmigiani S, Rubin BS, Schoenfelder G, Sonnenschein C, Soto AM, Talsness CE, Taylor JA, Vandenberg LN, Vandenbergh JG, Vogel S, Watson CS, Welshons WV, Zoeller RT (2009). Why public health agencies cannot depend on good laboratory practices as a criterion for selecting data: the case of bisphenol A. Environ Health Perspect.

[15] Rochester JR, Bolden AL (2015). Bisphenol S and F: a systematic review and comparison of the hormonal activity of bisphenol A substitutes. Environ Health Perspect.

